# Comprehensive profiling of extracellular RNA in HPV-induced cancers using an improved pipeline for small RNA-seq analysis

**DOI:** 10.1038/s41598-020-76623-z

**Published:** 2020-11-10

**Authors:** Fangjia Tong, Arlise Andress, Gongyu Tang, Ping Liu, Xiaowei Wang

**Affiliations:** 1 Department of Radiation Oncology, Washington University School of Medicine, St. Louis, MO USA; 2 Department of Pharmacology and Regenerative Medicine, University of Illinois at Chicago, Chicago, IL USA; 3 University of Illinois Cancer Center, Chicago, IL USA

**Keywords:** Gene expression profiling, Tumour virus infections

## Abstract

Extracellular RNAs (exRNAs) have attracted great attention due to their essential role in cell-to-cell communication as well as their potential as non-invasive disease biomarkers. However, at present, there is no consensus on the best method to profile exRNA expression, which leads to significant variability across studies. To address this issue, we established an experimental pipeline for comprehensive profiling of small exRNAs isolated from cell culture. By evaluating six RNA extraction protocols, we developed an improved method for robust recovery of vesicle-bound exRNAs. With this method, we performed small RNA sequencing of exosomes (EXOs), microvesicles (MVs) and source cells from 14 cancer cell lines. Compared to cells, EXOs and MVs were similarly enriched in tRNAs and rRNAs, but depleted in snoRNAs. By miRNA profiling analysis, we identified a subset of miRNAs, most noticeably miR-122-5p, that were significantly over-represented in EXOs and MVs across all 14 cell lines. In addition, we also identified a subset of EXO miRNAs associated with cancer type or human papillomavirus (HPV) status, suggesting their potential roles in HPV-induced cancers. In summary, our work has laid a solid foundation for further standardization on exRNA analysis across various cellular systems.

## Introduction

Extracellular vesicles (EVs) are lipid-bilayer particles that are released from many types of cells^1,2^. Two major classes of EVs are commonly studied, including exosomes (EXOs) and microvesicles (MVs)^3^. EXOs are generated by fusing the multivesicular body to the plasma membrane, and MVs are released directly from cell membrane^4^. Recently, it has been demonstrated that EVs play essential roles in cross-cell communications, especially for immune response and tumor progression^5–9^. Recent studies have reported that EVs can regulate the bioactivities of recipient cells by transferring RNA cargo, such as miRNAs and mRNAs^10,11^. At present, extracellular RNAs (exRNAs) carried by EVs are intensely studied due to their important regulatory roles in cell-to-cell communications as well as their potential as non-invasive disease biomarkers^12,13^.

Although there have been many studies focusing on exRNAs, one major obstacle in this emerging field is the lack of robust methods for exRNA isolation and expression profiling analysis. For a typical EV-related cell biology study, as the first step, it is important to isolate EVs of high purity from cell culture medium. To date, differential ultracentrifugation is regarded as the gold standard for EV isolation, producing the highest purity compared with other alternative methods^14–17^. As the second step, extraction of exRNAs is usually required for further expression profiling analysis. To this end, various methods have been proposed to extract exRNAs, leading to significant variability across different studies. Thus, it is important to establish a robust standardized exRNA extraction method that can be broadly applied to most cell biology studies^18,19^. To address this challenge, we developed an improved pipeline for exRNA isolation and small RNA-seq profiling analysis. Using this pipeline, we performed small RNA-seq for EXOs, MVs and source cells from 14 cancer cell lines. In this way, we were able to comprehensively identify common as well as cell specific exRNA expression profiles in human papillomavirus (HPV)-induced cancers.

## Results

### Establishing an improved method for exRNA extraction

Previous studies have utilized glycogen as an inert carrier to enhance RNA extraction yield^20,21^. Thus, we evaluated the impact of glycogen addition during exRNA extraction. We selected eight miRNAs ranging from low to high expression in EVs: miR-126-3p, miR-191-5p, miR-27a, miR-26-5p, miR-21-5p, miR-24-3p, let-7a-5p and miR-92-3p. The expression of these miRNAs was detected by real-time RT-PCR. As shown in Supplementary Figure S1, the addition of glycogen during exRNA extraction resulted in increased yield for all tested miRNAs, as indicated by an average decrease of 1.92 Ct values. Thus, we decided to include glycogen carrier in all extraction protocols.

**Figure 1:**
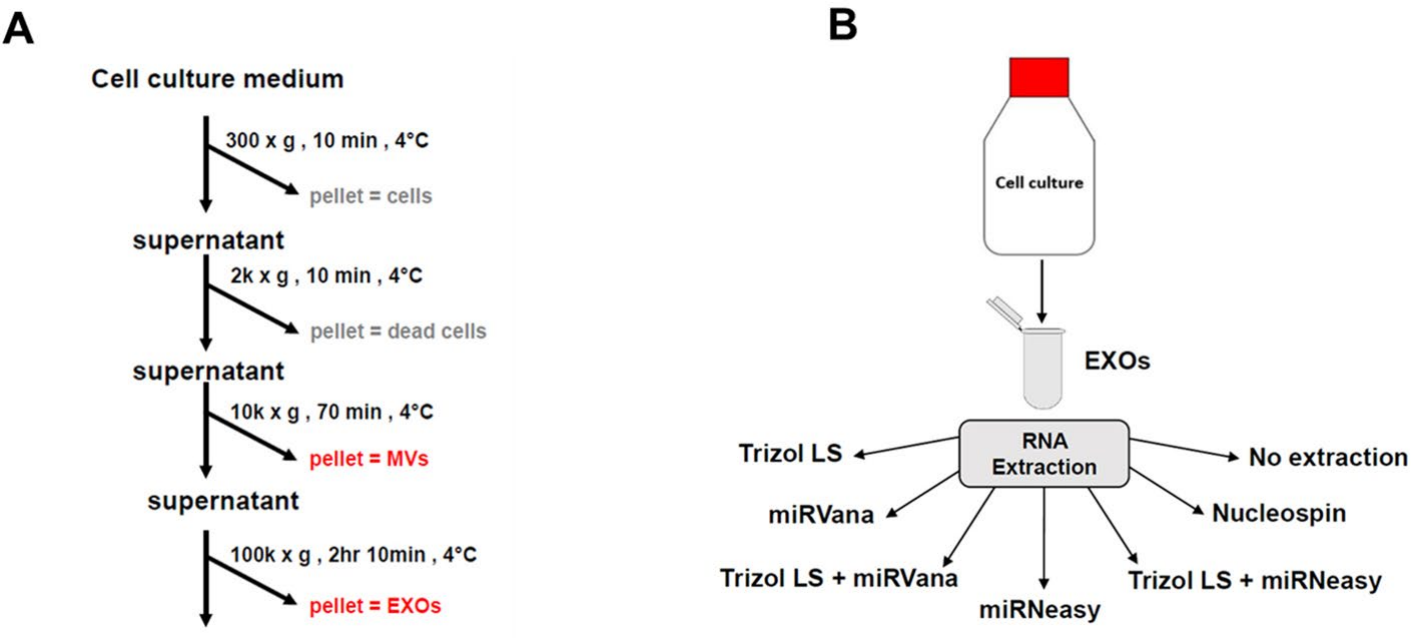
Sample preparation flow chart. (**A**) Flowchart showing the times and speeds of centrifugation for EV isolation. (**B**) EVs were isolated from cell culture medium and then aliquoted for RNA extraction by six different methods.

**Figure 2:**
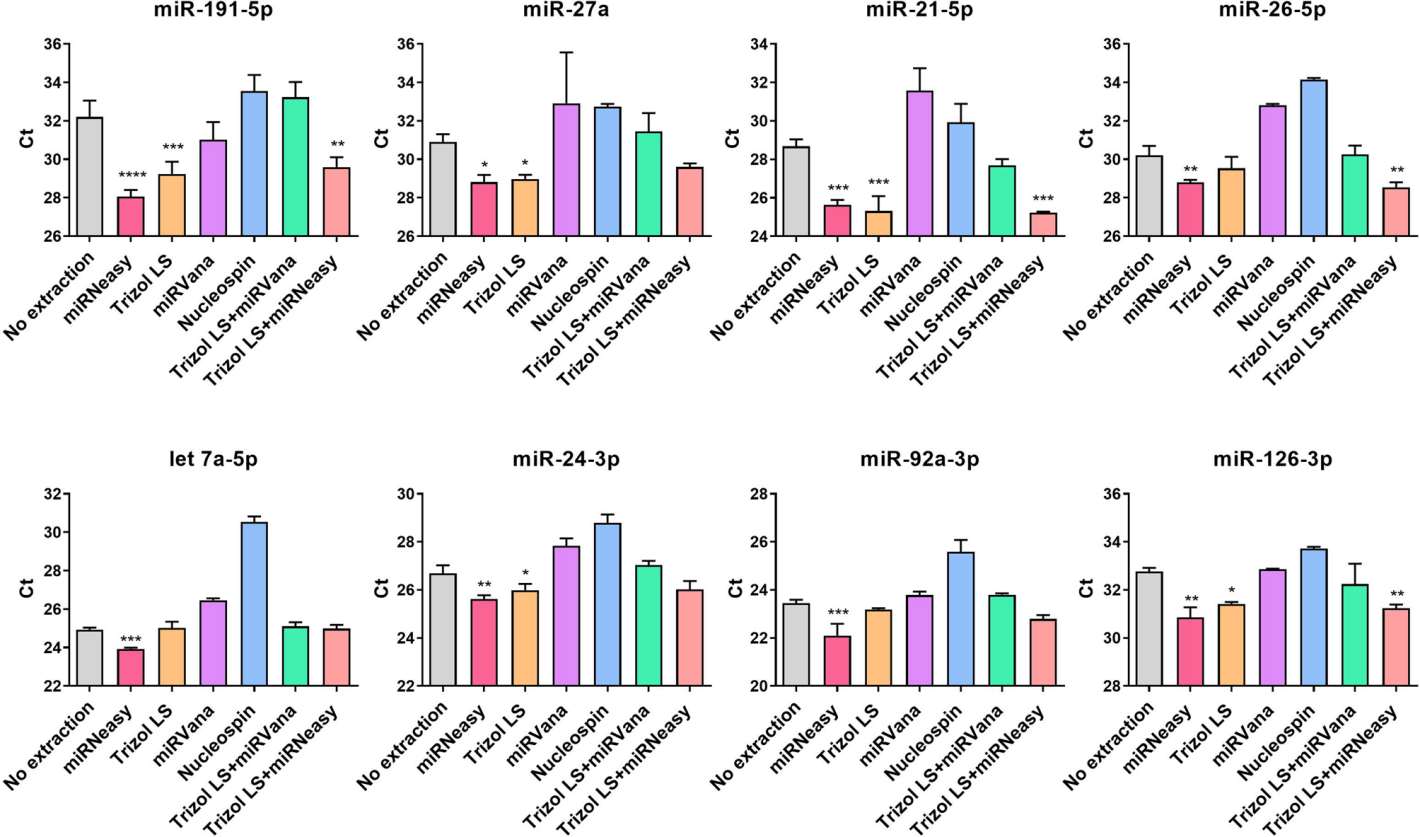
Comparison of EXO miRNA yield by real-time RT-PCR. Average Ct values were presented for eight individual miRNAs. Quantification of miRNA was performed by real-time RT-PCR. The mean values ± SD of three independent experiments are shown. *P < 0.05, **P < 0.01, ***P < 0.001.

EXOs were isolated from the cell culture medium and split into aliquots for RNA extraction by six different methods (Fig. 1B). These methods were also compared with no extraction control for RNA yield by real-time RT-PCR. The results showed that the six methods had drastically different RNA yields as evaluated by Ct values for selected miRNAs (Fig. 2). Specifically, miRNeasy had the best overall performance as indicated by the lowest Ct values for seven of the eight miRNAs. Trizol LS had the second-best performance. miRVana and Nucleospin mostly showed higher Ct values compared with no extraction control. To assess whether combining methods leads to further improvement, we tested two combinations of miRNeasy, Trizol LS and miRVana. The combination of Trizol LS and miRVana was better than miRVana, but worse than Trizol LS alone; the combination of Trizol LS and miRNeasy resulted in higher yield of miRNAs than Trizol LS, but still slightly lower yield than miRNeasy alone. Furthermore, we also compared the yields of these methods for miRNA isolation from MVs. Similarly, miRNeasy had the best yield for MV miRNAs (Supplementary Fig. S2). In summary, among the six methods we evaluated, miRNeasy supplemented with glycogen carrier had the best yield for miRNA extraction.

To evaluate technical variability of the miRNeasy/glycogen extraction method, we performed correlative analysis of miRNA expression profiles. Specifically, we constructed small RNA-seq libraries for exRNAs isolated from EXOs. As shown in Supplementary Figure S3, the miRNeasy/glycogen method showed excellent reproducibility, as indicated by very high correlation coefficient between technical duplicates (R^2^ value of 0.9974).

### EXOs, MVs and cells had distinct small RNA expression profiles

In our study, we decided to focus on HPV-related cancers, including both head and neck squamous cell carcinoma (HNSCC) and cervical squamous cell carcinoma (CSCC). HPV is an important risk factor for both HNSCC and CSCC^22^. The characteristics of the cell lines included in our study are listed in Table 1. To confirm the HPV status in the 14 cell lines, we performed real-time RT-PCR to determine the expression of HPV oncogenes E6 and E7. Among the HNSCC cell lines, SCC-154, SCC-47 and SCC-090 showed HPV-16 E6 and E7 transcriptional activity (Supplementary Fig. S4A); among the CSCC cell lines, SiHa and Caski were HPV-16 positive whereas SW756, C4I and Hela were HPV-18 positive (Supplementary Fig. S4B). The rest of the cell lines were HPV negative as determined by HPV E6 and E7 assays.

**Table 1:** Characteristics of the cell lines used in this study.

Cell line	Cancer type	HPV type
SCC-154	HNSCC	HPV-16
SCC-090	HNSCC	HPV-16
SCC-047	HNSCC	HPV-16
UPCI-017	HNSCC	Negative
UPCI-068	HNSCC	Negative
SCC-4	HNSCC	Negative
SCC-1	HNSCC	Negative
SiHa	CSCC	HPV-16
HeLa	CSCC	HPV-18
Caski	CSCC	HPV-16
C4I	CSCC	HPV-18
SW756	CSCC	HPV-18
HT-3	CSCC	Negative
C33A	CSCC	Negative

**Figure 3:**
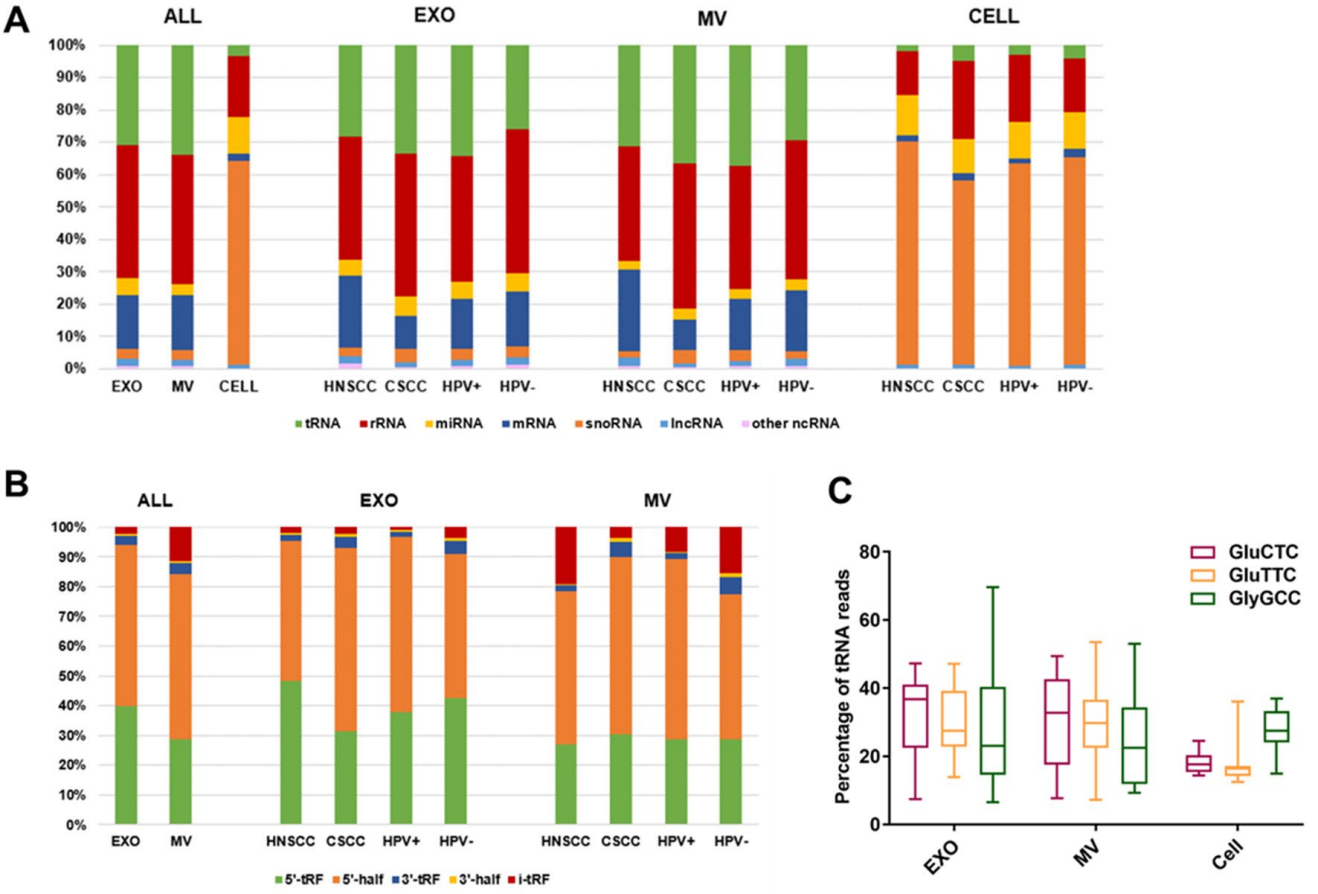
The small RNA composition of EXOs, MVs and cells. (**A**) The small RNA composition in all samples combined. (**B**) The tRNA composition of EXOs and MVs. (**C**) The percentage of three most abundant tRNA species in EXOs, MVs and cells.

We then performed small RNA-seq for EXOs, MVs and the source cells from these 14 cell lines. We determined the RNA composition by counting the percentages of reads mapped to the following RNA species: mRNA, miRNA, tRNA, ribosomal RNA (rRNA), long non-coding RNA (lncRNA), small nucleolar RNA (snoRNA) and other non-coding RNA (ncRNA) (Fig. 3). Results from all 14 cell lines were combined to compare the percentages of these RNA species across EXOs, MVs and cell lines. As shown in Fig. 3A, rRNA was the most abundant species in EXOs and MVs, representing 41% and 40% of total exRNA reads, respectively. The percentage of tRNA was also much higher in EXOs and MVs when compared with cells (31% and 34% vs. 3%). Similarly, mRNA was significantly enriched in EXOs and MVs as compared with cells (16% and 17% vs. 2%). On the other hand, snoRNA was most abundant in cells, while only representing a small fraction of total exRNA in EXOs and MVs (63% vs. 3% and 3%). The percentage of miRNA was the highest in cells, representing 11% of total reads. As to exRNAs, miRNA was more enriched in EXOs compared with MVs (5% and 3%, respectively).

Next, we evaluated potential differences in RNA composition that could be related to cancer type or HPV status. When the EXOs were stratified by cancer type, the percentage of mRNA was higher in HNSCC compared with CSCC (22% vs. 10%). On the other hand, no significant difference in RNA composition was observed between HPV+ EXOs and HPV− EXOs. The results of MV expression analysis were similar to that of EXOs. For the cell component, the percentage of snoRNA was slightly higher in HNSCC compared with CSCC, but the difference was not statistically significant. When stratified by HPV status, there was no significant difference between HPV+ and HPV− cells in small RNA composition. Overall, the RNA compositions for EXO and MV closely resembled each other, both of which were drastically different from that of cells.

As described above, tRNAs were significantly enriched in EXOs and MVs. We further compared the tRNA composition in EXOs, MVs and cells. As shown in Fig. 3B, tRNA 5′-half was the most abundant in both EXOs and MVs, representing 54% and 56% of total tRNA reads, respectively. On the other hand, 5′-tRNA fragment (5′-tRF) was more enriched in EXOs while internal-tRF (i-tRF) was more enriched in MVs. Next, we evaluated potential differences in tRNA composition that could be related to cancer type or HPV status. When EXOs and MVs were analyzed separately, no significant difference was observed across cancer type or HPV status (Fig. 3B).

By comparing tRNA species across all samples, we found that GluCTC, GluTTC and GlyGCC were the most abundant (Fig. 3C). Among them, GluCTC was the most overrepresented tRNA in EXOs and MVs (32.6% and 29.9%, respectively) while GlyGCC was the most overrepresented one in cells (27.6%). Thus, our results showed that EXOs and MVs shared similar characteristics in tRNA composition, but not with cells.

### EXOs and MVs shared similar miRNA expression profiles

**Figure 4:**
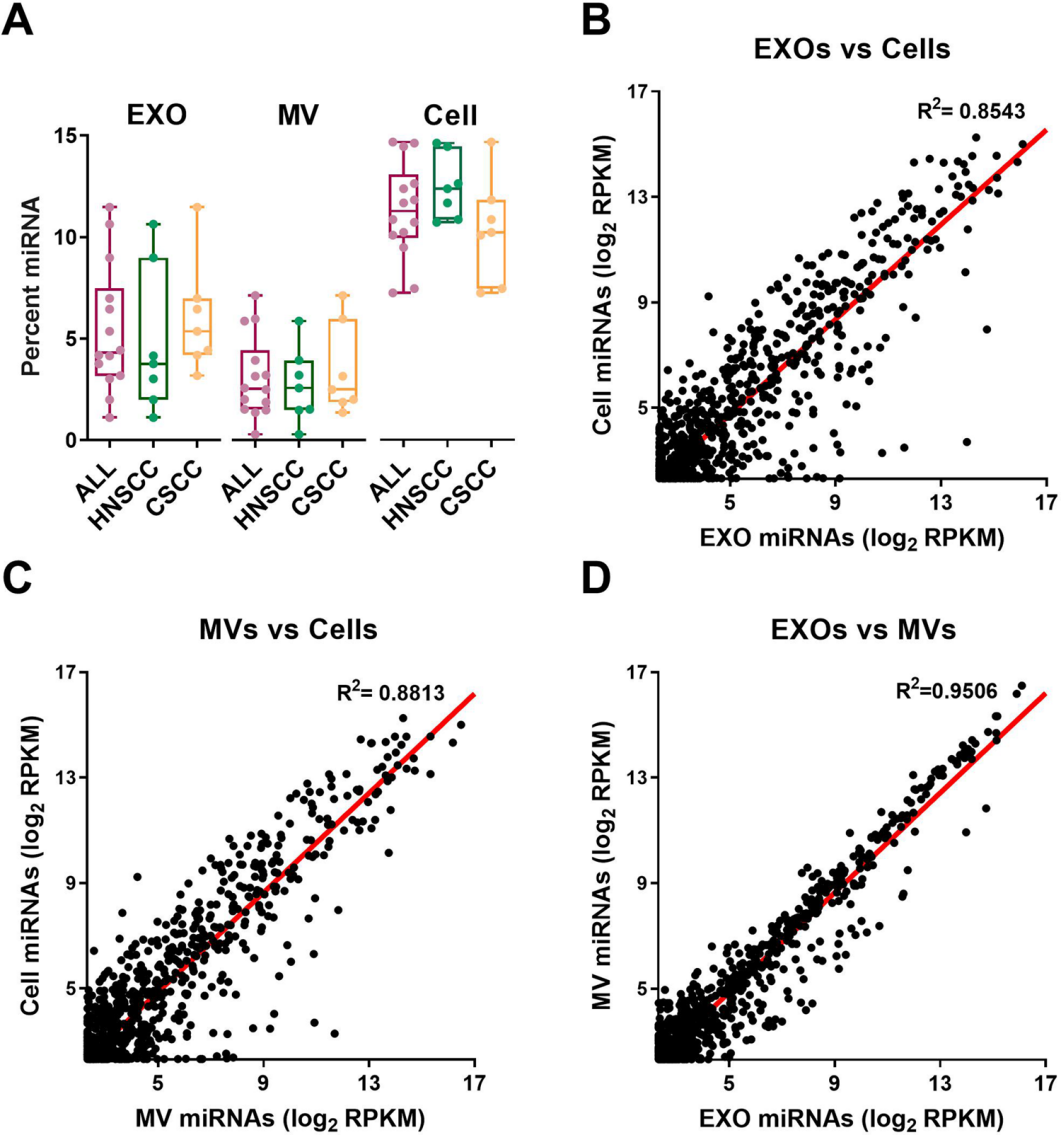
The miRNA profiles of EXOs, MVs and source cells. (**A**) The percentage of miRNAs in EXOs, MVs and cells. (**B**–**D**) Scatter plots for miRNA expression correlation between EXOs and cells (**B**), between MVs and cells (**C**), and between EXOs and MVs (**D**).

EXO miRNAs from cancer cells could potentially serve as non-invasive biomarkers for disease diagnosis and prognosis. We analyzed the percentages of miRNA reads among total small RNA-seq reads in EXOs, MVs and source cells. As shown in Fig. 4A, miRNAs were significantly more represented in cells when compared with EXOs and MVs (*P* < 0.001). Among the 14 cell lines, we did not observe any significant difference in the percentage of total miRNA reads across cancer type. We further compared miRNA expression profiles of EXOs, MVs and source cells. Compared with cells, the miRNA profiles of EXOs and MVs were both distinctly different, with R^2^ of 0.8543 and 0.8813, respectively (Fig. 4B,C). On the other hand, EXOs and MVs shared similar miRNA profiles, with R^2^ of 0.9506 (Fig. 4D).

### miRNAs that were selectively enriched in EXOs and MVs

**Figure 5:**
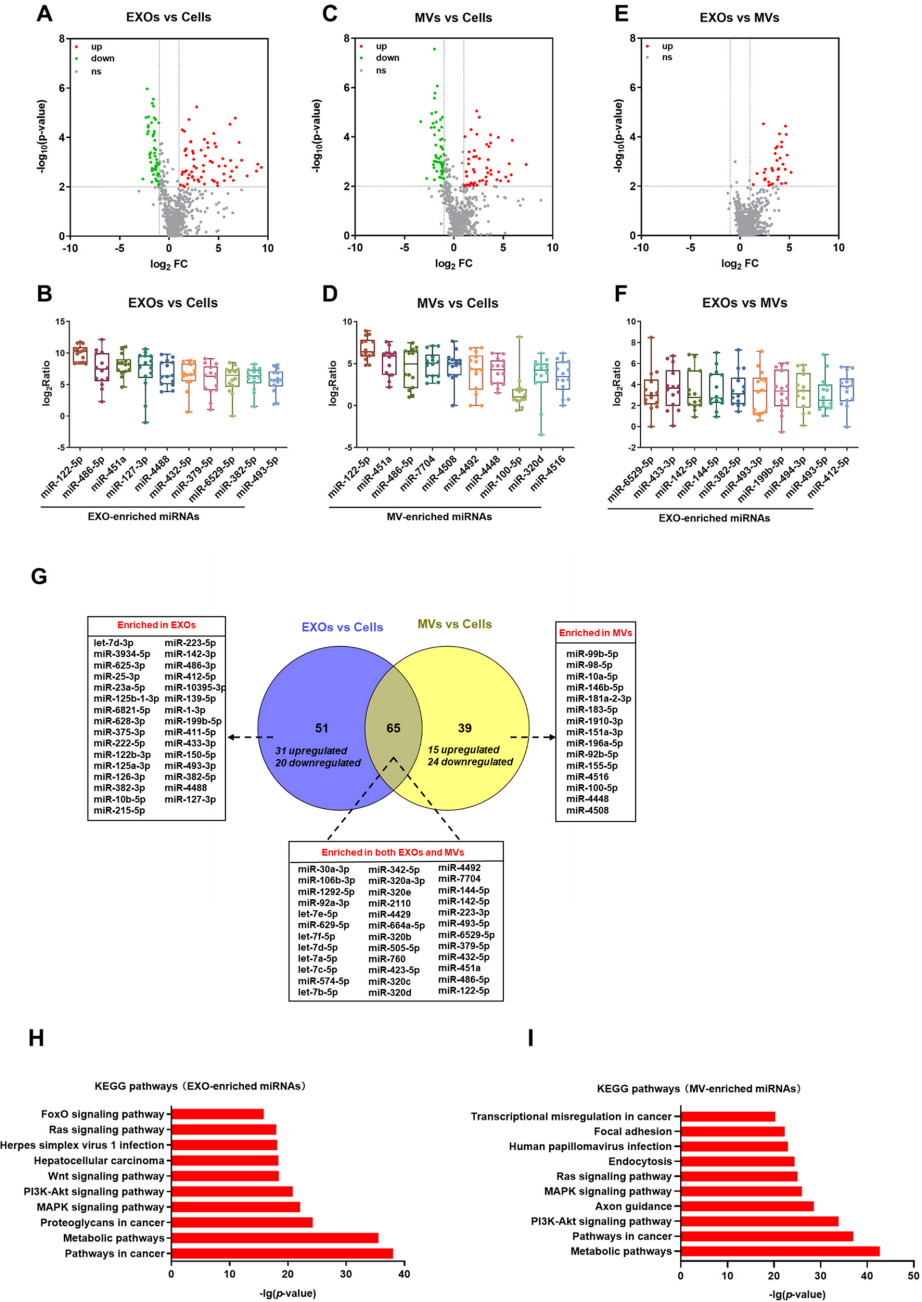
miRNAs enriched in EXOs and MVs. Volcano plots for differentially expressed miRNAs between EXOs and cells (**A**), between MVs and cells (**C**), and between EXOs and MVs (**E**). The expression of top 10 differentially expressed miRNAs between EXOs and cells (**B**), between MVs and cells (**D**), and between EXOs and MVs (**F**). (**G**) Venn diagram to summarize differentially expressed miRNAs. (**H**) The top 10 signaling pathways involving EXO-enriched miRNAs. (**I**) The top 10 signaling pathways involving MV-enriched miRNAs.

We compared differentially expressed miRNAs among EXOs, MVs and their source cells. Altogether, 116 miRNAs (67 upregulated and 49 downregulated) were differentially expressed between EXOs and cells in the 14 cell lines (Fig. 5A; ten most significant EXO-enriched miRNAs shown in Fig. 5B). Similarly, 104 miRNAs (51 up-regulated and 53 down-regulated) were differentially expressed between MVs and cells in the 14 cell lines (Fig. 5C; top ten MV-enriched miRNAs shown in Fig. 5D). Of note, miR-122-5p was the most enriched miRNA, over-represented by 1384 and 159 fold on average in EXOs and MVs, respectively. Several other miRNAs, including miR-486-5p, miR-451a and miR-4488, were also highly enriched in EXOs and MVs (Fig. 5B,D). When EXOs and MVs were compared directly, 35 miRNAs were differentially expressed, all of which were enriched in EXOs (Fig. 5E; ten most significant miRNAs listed in Fig. 5F). Previous studies suggest that the mechanisms for miRNA packaging in EXOs and MVs are distinctively different^23^. Thus, we further investigated the commonality and differences of the miRNA profiles between EXOs and MVs. To this end, we focused on 155 miRNAs that were differentially expressed in EXOs and/or MVs as compared to cells. As shown in Fig. 5G, 65 miRNAs (including 36 enriched ones) were commonly found in both EXOs and MVs. In comparison, 31 and 15 miRNAs were enriched in either EXOs or MVs, but not both. To further investigate the signaling pathways that could be affected by EV-enriched miRNAs, predicted target genes of EXO- and MV-enriched miRNAs were entered into the KEGG enrichment analysis, respectively. The top 10 significant pathways were shown in Fig. 5H,I. Both EXO- and MV-enriched miRNAs were significantly enriched in “Pathways in cancer”, “Metabolic pathways”, “PI3K-Akt signaling pathway”, “MAPK signaling pathways” and “Ras signaling pathways”.

### Cancer type-related miRNAs in EXOs and cells

**Figure 6:**
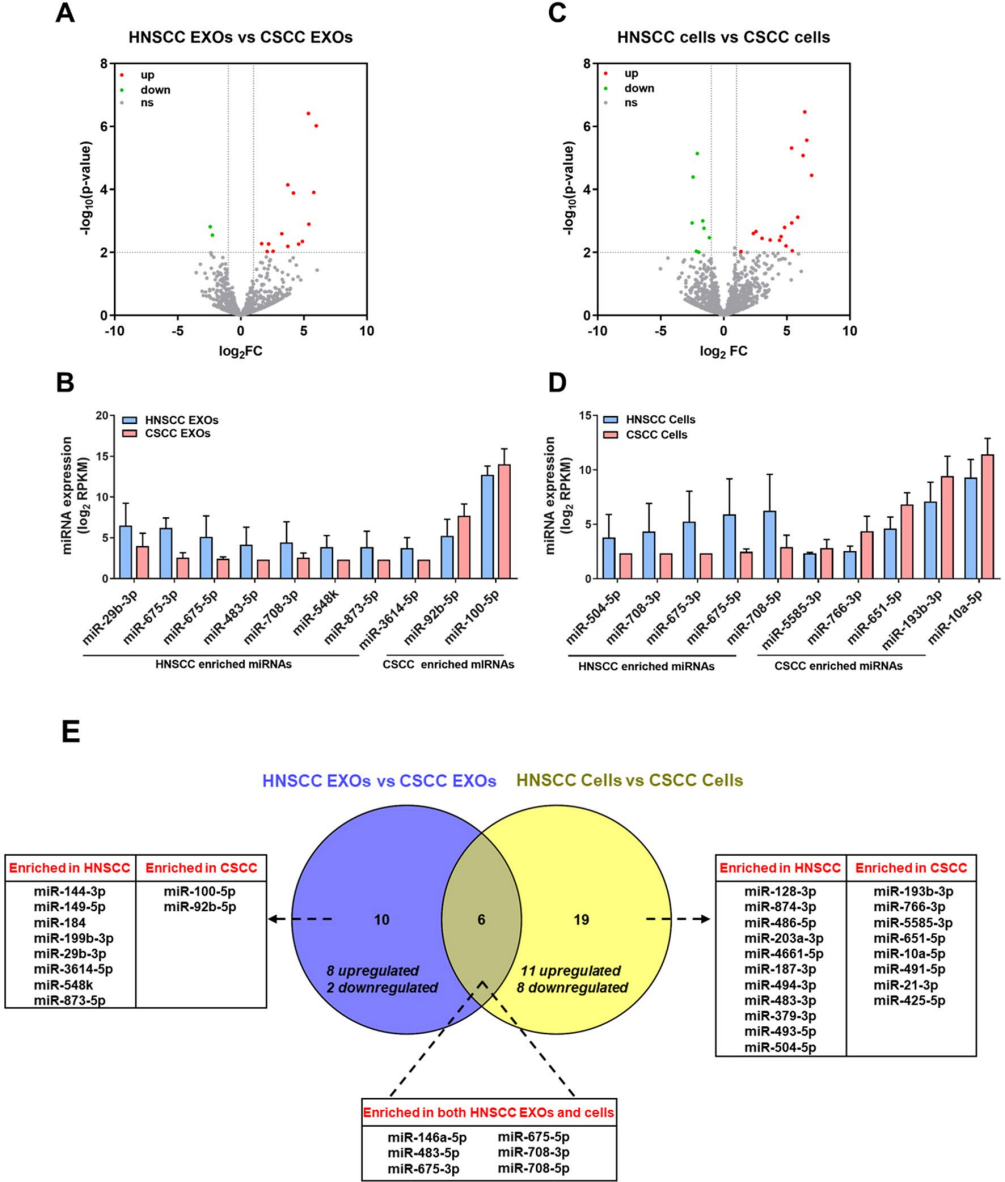
Cancer type-related miRNAs in EXOs and cells. (**C**,**C**) Volcano plots for differentially expressed miRNAs between HNSCC and CSCC EXOs (**A**), and between HNSCC and CSCC cells (**C**). (**B**,**D**) The expression of top 10 differentially expressed miRNAs between HNSCC and CSCC EXOs (**B**), and between HNSCC and CSCC cells (**D**). (**E**) Venn diagram to summarize differentially expressed miRNAs.

Next, we focused on identifying EXO miRNAs that were correlated to specific cancer types (HNSCC vs. CSCC). A total of 16 miRNAs were found to be differentially expressed (Fig. 6A). Among them, 14 were enriched in HNSCC EXOs while two were enriched in CSCC EXOs (ten most significant miRNAs listed in Fig. 6B). We also analyzed differentially expressed miRNAs between HNSCC and CSCC cells. A total of 26 miRNAs were significantly different, with 17 enriched in HNSCC cells and eight enriched in CSCC cells (Fig. 6C; ten most significant miRNAs listed in Fig. 6D). Next, by comparing the miRNA lists described above, we identified six miRNAs that were enriched in both EXOs and cells of HNSCC as compared to CSCC (Fig. 6E). When focusing on the EXO component, eight miRNAs were specifically upregulated in HNSCC while two were upregulated in CSCC (Fig. 6E).

### HPV-related miRNAs in EXOs and cells

**Figure 7:**
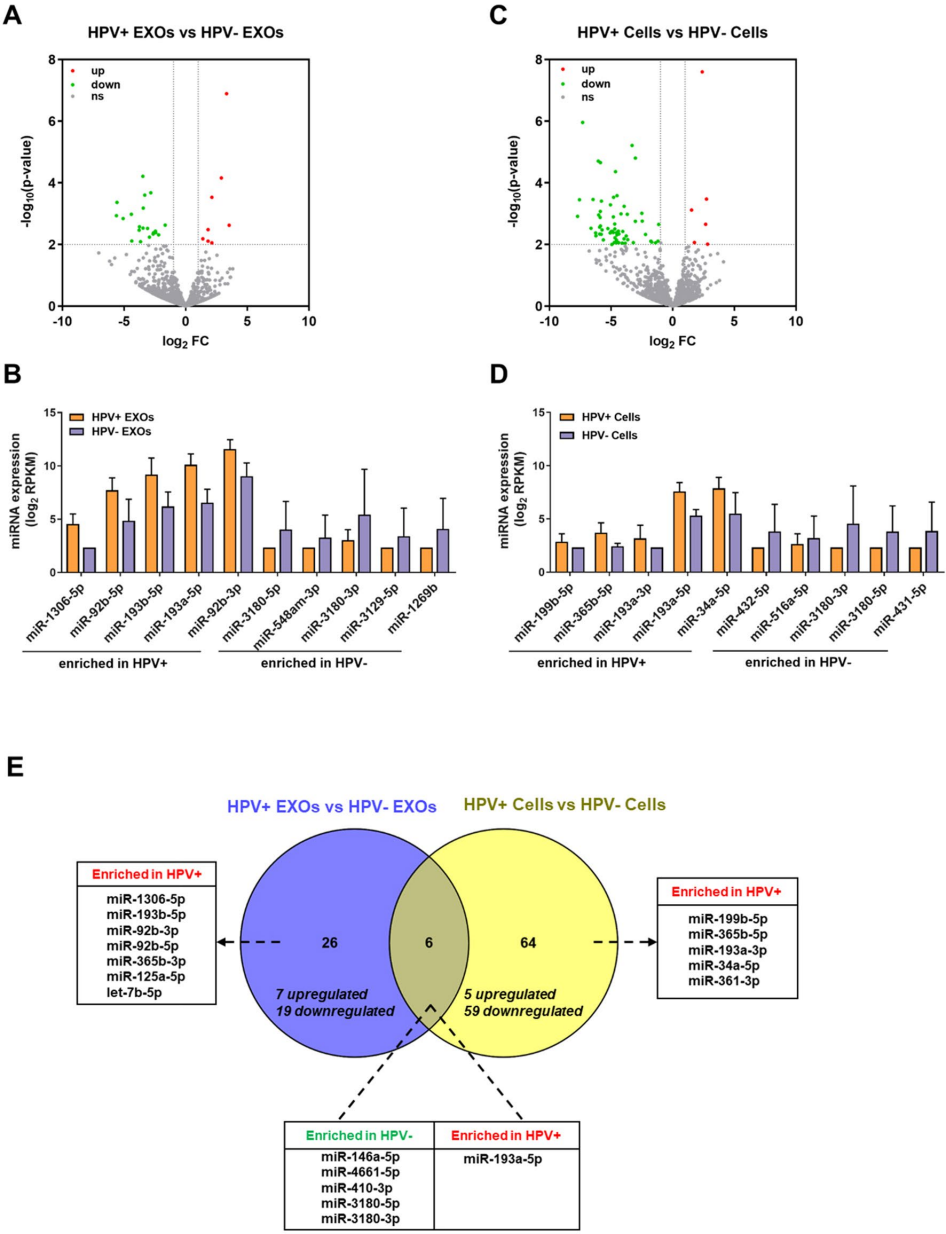
HPV-related miRNAs in EXOs and cells. (**A**,**C**) Volcano plots for differentially expressed miRNAs between HPV+ and HPV− EXOs (**A**) and between HPV+ and HPV− cells (**C**). (**B**,**D**) The expression of top 10 differentially expressed miRNAs between HPV+ and HPV− EXOs (**B**), and between HPV+ and HPV− cells (**D**). (**E**) Venn diagram to summarize differentially expressed miRNAs.

Next, we investigated HPV-related miRNAs in EXOs and cells. A total of 32 miRNAs were differentially expressed between HPV+ and HPV− EXOs (Fig. 7A). Among them, eight were enriched in HPV+ EXOs while 24 were enriched in HPV− EXOs. Five most upregulated and five most downregulated miRNAs were listed in Fig. 7B. In a similar way, 70 miRNAs were found to be differentially expressed between HPV+ and HPV− cells, of which six were enriched in HPV+ cells and 61 were enriched in HPV− cells (Fig. 7C; top 10 miRNAs listed in Fig. 7D). We further cross-referenced these miRNA lists and identified six miRNAs that were associated with HPV status in both EXOs and cells (Fig. 7E). Of the six, miR-193a-5p was enriched in HPV+ EXOs and cells while miR-146a-5p, miR-4661-5p, miR-410-3p, miR-3180-5p and miR-3180-3p were enriched in HPV− EXOs and cells. In the same way, we also identified 26 and 64 HPV-associated miRNAs in EXOs or cells, but not both (Fig. 7E).

## Discussion

Compared with source cells, EXOs and MVs were both highly enriched in tRNAs and rRNAs, but depleted in snoRNAs. Among all tRNA species in exRNAs, 5′-tRFs and 5′-halves were most abundant, representing about 90% of total tRNA reads. Similar observations on tRNA composition of exRNAs were reported previously based on a limited number of cell lines^1,24,25^. Our comprehensive analysis of 14 cell lines further indicates that significant enrichment of 5′-tRFs and 5′-halves in exRNAs is likely a persistent phenomenon across most human cell lines.

The most recognized function of tRNAs is to act as key adaptor molecules for peptide elongation during translation. In addition, recent studies have demonstrated that tRNA-derived fragments may play a variety of regulatory roles for gene expression control at the post-transcriptional level^26,27^. These fragments can be broadly classified into two major groups: tRNA halves and tRFs. Dysregulation of their biogenesis processes may potentially lead to various human diseases^28^, and the expression profiles of tRNA fragments could be used as non-invasive disease biomarkers, as reported by multiple studies^29–32^. Further studies have shed light on the mechanisms underlying the biogenesis of tRNA fragments from mature intact tRNAs^33^. Thus, the highly enriched tRNA fragments in exRNAs may have important functional roles in cell-to-cell communications.

In contrast to tRNA fragments, snoRNAs were drastically depleted in exRNAs as compared to cells. snoRNAs are small ncRNAs abundantly expressed in eukaryotic nuclei that mediate the maturation of rRNAs^34,35^. Our results indicate that snoRNAs represent over 60% of all small RNA reads from cells. However, there was little presence of snoRNAs in exRNAs from any of the 14 cell lines. Thus, the specific mechanisms for exRNA transport effectively excluded snoRNAs from packaging into EVs.

The miRNA component in EVs has been intensely studied in recent years, and many studies indicate that EV miRNAs act as inter-cell communicators in a variety of biological processes and diseases especially in cancer. However, previous studies mainly focused on the analysis of miRNA profiles in EXOs. In contrast, by comprehensive comparison of miRNA profiles in EXOs, MVs and source cells from 14 cell lines, we revealed that the miRNA profiles of EXOs and MVs were very similar, both of which were distinctly different from that of cells. Thus, both EXOs and MVs could be useful for studying the roles of miRNAs in inter-cell communication.

EXO and MV constitute two main subclasses of EV. EXOs are formed during multivesicular body fusion with plasma membrane, whereas MVs are released directly from the plasma membrane. Due to different mechanisms in biogenesis, EXOs and MVs may carry different miRNA cargo. Indeed, we observed that multiple miRNAs were differentially expressed when MVs and EXOs were compared directly. Despite the differences, MVs and EXOs showed largely similar miRNA profiles, especially when compared with source cells. Most strikingly, miR-122-5p was overrepresented by over 100 fold in both EXOs and MVs from all 14 cell lines. This result suggests that miR-122-5p may potentially play a major role in inter-cell communication in cancers. As supporting evidence, miR-122-5p can promote tumor metastasis via EXO transportation in breast cancer^36^ and exosomal miR-122-5p level is related to chemosensitivity in liver cancer^37^. Our analysis indicates that high miR-122-5p expression in EVs is likely a general mechanism for cell-to-cell communication in cancers.

The enrichment of miR-122-5p could be explained by the specific mechanisms for miRNA sorting into EVs. There are four alternative pathways for sorting miRNAs into EVs, including nSMase2-dependent pathway, hnRNPs-dependent pathway, miRNA 3′-end sequence dependent pathway and miRISC-guided pathway^23,38^. Through these pathways, functional miRNAs are sorted into EVs to regulate cell-to-cell communications. Thus, differential expression of specific miRNAs in EVs is likely related to differences in the sorting mechanisms, which warrants further investigation in the future.

By comparing subsets of the 14 cell lines, we have also identified miRNAs in EVs that are related to specific cancer type or HPV status. To date, most studies focused on EV miRNA profiles in one specific type of cancer, and few studies made comparison across different cancer types. Here, we compared miRNA expression between HNSCC and CSCC EXOs, and identified cancer type-related miRNAs in EXOs. Among them, miR-29b-3p was enriched in HNSCC EXOs. It would be interesting to further elucidate the mechanisms underlying differential sorting of EV miRNAs in different cancers.

Previous studies have revealed that HPV may regulate miRNA expression in both EVs and source cells^39,40^. To further investigate the association between HPV and miRNA expression, we analyzed 14 cell lines, eight of which are HPV positive (including three HNSCC and five CSCC cell lines). In this way, we identified a subset of HPV-related miRNAs in EXOs. Among them, miR-92b-5p and miR-146a-5p were upregulated in HPV+ and HPV− EXOs, respectively. Consistently, a recent study analyzed exosomal miRNA profile of HeLa cells, reporting enrichment of miR-92b-5p in HPV+ EXOs^41^.

miRNAs have been identified to play important roles in both EVs and cells^1,12^. In our study, miR-223-3p and miR-423-5p were observed enriched explicitly in HNSCC EVs. Previous studies examined the plasma levels of these miRNAs and found high expression of these miRNAs in HNSCC patients^42,43^. For CSCC EV-enriched miRNAs, let-7d-3p, miR-144-5p and miR-10b-5p were highly expressed^44^. Consist with previous studies, these miRNAs were found enriched in plasma of CSCC patients. In tumor cells, miR-504-5p is an important oncogenic miRNA that is involved in HNSCC. High level of miR-504-5p was correlated with advanced tumor stage^45^. In our study, miR-504-5p was observed enriched in HNSCC cells. For CSCC, miR-10a-5p was reported overexpressed in tumor cells with lymph node metastasis^46^. In our study, miR-10a-5p was found highly expressed in CSCC cells. In order to investigate the potential functions of enriched miRNAs, we performed KEGG enrichment analysis to explore associated signaling pathways. We found these enriched miRNAs were significantly associated with “Pathways in cancer”, “Metabolic pathways” and “PI3K-Akt signaling pathway”. This result suggests that these enriched miRNAs may have functional roles in carcinogenesis process and tumor development. The functions of these miRNAs in HPV-induced cancers warrant further investigation.

It should be noted that our study is limited by the purity of isolated EXOs and MVs. In EV studies, differential ultracentrifugation is regarded as the gold standard for EV isolation. However, due to the overlapping size distributions of isolated EXOs and MVs, it is not possible to completely separate these two groups. Although we isolated the EVs according to standard protocols, the EVs that we isolated should be considered as EXO- or MV-enriched vesicles.

In summary, we have developed an improved pipeline for exRNA profiling studies. With this pipeline, we performed small RNA-seq for EXOs, MVs and source cells from 14 cancer cell lines and identified specific miRNAs that were significantly over-represented in EVs. Our work has laid a foundation for further standardization on exRNA analysis across various cellular systems.

## Methods

### Cell culture and preparation of EV-free FBS

To obtain EV-free FBS, FBS (Gibco) was ultracentrifuged at 120,000×*g* for 20 h at 4 °C. The supernatant was then filtered through 0.22 µm filter (Merck Millipore). Human head and neck squamous cell carcinoma (HNSCC) cell lines SCC-090, SCC-047, SCC-1, SCC-4, UPCI-017 and UPCI-068 were cultured in DMEM containing 10% EV-free FBS and 0.1 mM MEM nonessential Amino acids (Gibco). Human HNSCC cell line SCC-154 was cultured in MEM containing the same supplements as described above. Human cervical squamous cell carcinoma (CSCC) cell line SiHa was cultured in DMEM containing 10% EV-free FBS. CSCC Caski cell line was cultured in RPMI-1640 containing 10% EV-free FBS. The remaining CSCC cell lines (Hela, SW756, HT-3, C33A and C4I) were cultured in iMDM containing 10% EV-free FBS.

### EV isolation

All the cells were cultured at 37 °C in a humidified atmosphere of 5% CO_2_. 5 ml cell culture medium from each cell line was used for EXO and MV isolation using the differential ultracentrifugation method. The times and speeds of centrifugation are shown in Fig. 1A. In brief, cell culture medium was centrifuged at 300×*g* for 10 min and 2000×*g* for 30 min sequentially to remove cells and debris, respectively. Then, the supernatant was centrifuged at 10,000×*g* for 70 min to collect MVs. The MV-depleted supernatant was then ultracentrifuged at 120,000×*g* for 70 min to pellet EXOs (all steps were performed at 4 °C). The EXO pellet was resuspended using PBS and subsequently aliquoted for RNA extraction.

### Nanoparticle tracking analysis (NTA)

The EVs were identified by ZetaView PMX 110 (Particle Metrix, Meerbusch, Germany) and corresponding software ZetaView 8.04.02. EVs were appropriately diluted with 1X PBS buffer. NTA measurement was recorded at 11 positions. The ZetaView software was utilized to quantify the concentration of the samples (particles/mL) and size distribution (in nanometer). Each sample was measured in triplicates. The size and concentration of EVs from SiHa cells were shown as an example in Supplementary Figure S5.

### exRNA extraction

exRNA was isolated using the following kits individually or in combination: Trizol LS (Invitrogen), miRVana miRNA Isolation (Ambion), miRNeasy Micro (Qiagen), and Nucleospin miRNA Plasma (Takara).

#### Trizol LS

First, 3× volumes of the Trizol LS reagent was added to the EVs and incubated for 5 min. Then, after adding 0.8× volume of chloroform, the mixture was incubated for 3 min and centrifuged for 15 min at 12,000×*g* at 4 °C. The RNA in the aqueous phase was precipitated by adding 2× volumes of isopropanol and 1 µg of glycogen (Thermo Fisher). The mixture was incubated for 10 min and then centrifuged for 10 min at 12,000×*g* at 4 °C. The RNA pellet was resuspended in 4× volumes of 75% ethanol and centrifuged for 5 min at 7500×*g* at 4 °C. After air drying for 10 min, RNA was eluted in 20 µL of RNase-free water.

#### miRVana miRNA kit

First, 0.6 ml of Lysis/Binding Buffer was added to the EVs and then 60 µL of miRNA Homogenate Additive was added. After incubating for 10 min on ice, equal volume of Acid-Phenol: Chloroform (Sigma) was added followed by centrifugation for 5 min at 10,000×*g*. The RNA in the aqueous phase was precipitated by adding 1.25× volumes of 100% ethanol and 1 µg of glycogen. Then, the mixture was loaded onto the Filter Cartridge and centrifuged for 15 s at 10,000×*g.* The column was then sequentially washed with 700 µL miRNA Wash Solution 1 and then twice with 500 µL Wash Solution 2/3. After final drying spin at 10,000×*g* for 1 min, the RNA was eluted in 20 µL pre-heated (95 °C) RNase-free water directly to the center of the filter and centrifuged for 1 min at 10,000×*g*.

#### miRNeasy Micro kit

First, 5× volumes of the QIAzol Lysis reagent was added to the EVs and incubated for 5 min. Then, equal volume of chloroform was added. The mixture was incubated for 3 min and centrifuged for 15 min at 12,000×*g* at 4 °C. The RNA in the aqueous phase was precipitated by adding 1.5× volumes of 100% ethanol and 1 µg of glycogen. The mixture was then loaded onto MinElute spin column and centrifuged at 8000×*g* for 15 s. The column was then sequentially washed with 700 µL Buffer RWT, 500 µL Buffer RPE and 500 µL 80% ethanol consecutively by centrifuging for 15 s at 8000×*g*. After final drying spin at 12,000×*g* for 5 min, RNA was eluted in 10 µL RNase-free water directly to the center of the spin column membrane and centrifuged for 1 min at 12,000×*g*.

#### Nucleospin

First, 90 µL of the MLP reagent was added to the EVs, incubated for 1 min and centrifuged for 3 min at 11,000×*g*. Next, 400 µL isopropanol and 1 µg glycogen were added. The mixture was loaded onto Nucleospin miRNA column, incubated for 2 min and centrifuged for 30 s at 11,000×*g*. Then, the column was consecutively loaded with 700 µL Buffer MW2, 250 µL Buffer MW2 and 50 µL rDNase, and incubated for 15 min. Then, 100 µL Buffer MW1, 700 µL Buffer MW2 and 250 µL Buffer MW2 were added to the Nucleospin miRNA column. After final drying spin at 11,000×*g* for 2 min, 20 µL RNase-free water was added to the center of the membrane. RNA was eluted by centrifuging for 1 min at 11,000×*g*.

#### Trizol LS + miRVana

First, 3× volumes of the Trizol LS reagent was added to the EVs and incubated for 5 min. Next, 0.8× volume of chloroform was added, the mixture was incubated for 3 min and centrifuged for 15 min at 12,000×*g* at 4 °C. The RNA in the aqueous phase was precipitated by adding 2× volumes of isopropanol and 1 µg of glycogen. Then, the mixture was loaded onto the Filter Cartridge and centrifuged for 15 s at 10,000×*g*. The column was then sequentially washed with 700 µL miRNA Wash Solution 1 and twice with 500 µL Wash Solution 2/3. After final drying spin at 10,000×*g* for 1 min, the RNA was eluted in 20 µL pre-heated (95 °C) RNase-free water directly to the center of the filter and centrifuged for 1 min at 10,000×*g*.

#### Trizol LS + miRNeasy

First, 3× volumes of the Trizol LS reagent were added to the EVs and incubated for 5 min. Then, 0.8× volume of chloroform was added, the mixture was incubated for 3 min and centrifuged for 15 min at 12,000×*g* at 4 °C. The RNA in the aqueous phase was precipitated by adding 2× volumes of isopropanol and 1 µg of glycogen. The mixture was then loaded onto MinElute spin column and centrifuged at 8000×*g* for 15 s. The columns were then washed with 700 µL Buffer RWT, 500 µL Buffer RPE and 500 µL 80% ethanol consecutively by centrifuging for 15 s at 8000×*g* between each washing step. After final drying spin at 12,000×*g* for 5 min, RNA was eluted in 10 µL RNase-free water directly to the center of the spin column membrane and centrifuged for 1 min at 12,000×*g*.

### Real-time RT-PCR to determine miRNA yield

The miRNA yield for extracted exRNA was analyzed with real-time RT-PCR. Specifically, quantification of eight miRNAs was performed, including miR-191-5p, miR-27a, miR-21-5p, miR-26-5p, miR-24-3p, miR-126-3p, miR-92-3p and let-7a-5p. The miRNA expression was detected as previously described^47^. In brief, reverse transcription (RT) was performed using the High Capacity cDNA Reverse Transcription kit (Applied Biosystems). Each 10 μL RT reaction included 1 μL of 10× RT buffer, 0.4 μL of 25× dNTP (100 mM), 0.4 μL of miRNA-specific RT primer mixture (250 nM), 0.5 μL of reverse transcriptase, 0.5 μL of RNase inhibitor and 10% of total exRNAs extracted by different methods. For no extraction control assay, incubation for 5 min at 75 °C was performed before RT reaction. Real-time PCR was performed with Power SYBR Green PCR master mix (Applied Biosystems). The Ct values were collected to compare the miRNAs yields from different methods.

### HPV assays

HPV assays were used to evaluate the expression of HPV E6 and E7 oncogenes, with detailed protocol described previously^48^. In brief, total RNA was extracted from each cell line with the miRVana kit. RT reaction was performed with the High Capacity cDNA Reverse Transcription kit. Real-time PCR was performed to quantify the cDNA product using power SYBR Green PCR master mix and 500 nM HPV-specific primers. Each HPV assay (E6 or E7 from HPV-16 and HPV-18) was individually performed on a 384-well PCR plate. The averaged expression levels of GAPDH and β-actin were used as the internal reference control for data normalization.

### Small RNA-seq and data analysis

Small RNA-seq libraries were constructed with the NEBNext small RNA-seq library preparation kit (New England Biolabs) according to manufacturer’s protocol with minor modifications. All reactions for exRNA libraries were performed with 1:8 diluted adapters and 18 cycles of PCR amplification. The library products were cleaned using Agencourt AMPure XP beads (Beckman Coulter), quantitated with dsDNA Quantifluor (Promega), and sequenced on Illumina HiSeq 3000 platform.

All raw reads were extracted from FASTQ files and identified 3′-adaptor sequences were trimmed. Processed reads were aligned to NCBI RefSeq transcriptome with Bowtie^49,50^ and then normalized using the RPKM method (reads per kilobase of transcript per million mapped reads). Specific for miRNA analysis, the reads were aligned to miRBase human miRNAs with Blat^51,52^. The aligned reads were then normalized to RPM counts (reads per million mapped miRNA reads), with a floor value of 5 established for lowly expressed miRNAs. Specific for tRNA analysis, all tRNA fragment (tRF) sequences were downloaded from MINTbase v2.0 and tRNA reads were mapped to tRFs using MINTmap pipeline^53,54^. Pearson’s correlation analysis was performed with the log_2_ transformed miRNA RPM reads to compare different methods or samples. Differentially expressed miRNAs were identified with paired t-test. Significant threshold cutoffs were set to log_2_ fold change ≥ 1 or ≤ − 1 with *p*-value ≤ 0.01. In addition, miRNA targets were predicted by miRDB^55^. KOBAS was used to identify the associated signaling pathways of the potential miRNA targets^56^.

## Supplementary information

Supplementary information.

## Data Availability

Raw sequencing data were deposited into the NCBI GEO database (accession GSE158659).
