# Supplementary material for: Comprehensive profiling of extracellular RNA in HPV-induced cancers using an improved pipeline for small RNA-seq analysis
Source: Sci Rep. 2020 Nov 10;10:19450. doi: 10.1038/s41598-020-76623-z (PMC7655837; doi:10.1038/s41598-020-76623-z)
Supplement: Supplementary file 1 — Supplementary information. [file 41598_2020_76623_MOESM1_ESM.pdf]

Supplementary Information For:

Comprehensive profiling of extracellular RNA in HPV-induced cancers using an improved pipeline for small RNA-seq analysis

Fangjia Tong[1], Arlise Andress[1], Gongyu Tang[1], Ping Liu[1] and Xiaowei Wang[1,2,3]

[1] Department of Radiation Oncology, Washington University School of Medicine, St. Louis, MO USA

[2] Department of Pharmacology and Regenerative Medicine, University of Illinois at Chicago

[3] University of Illinois Cancer Center, Chicago, IL USA

Corresponding author:

Xiaowei Wang, Phone: 312-413-2446; Email: xwang317@uic.edu

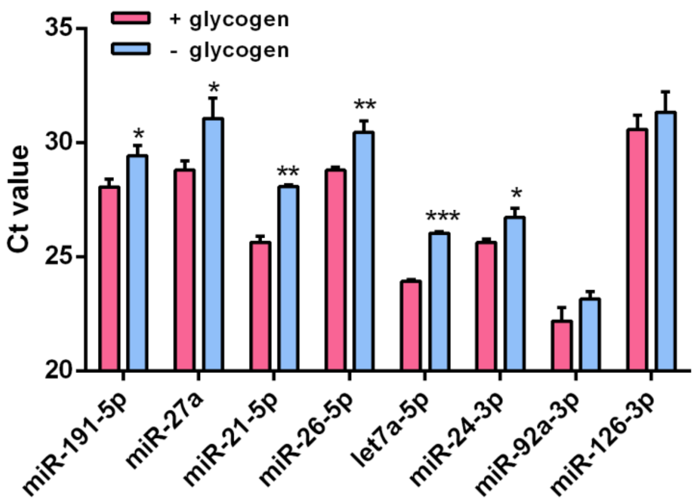

Supplementary Figure S1. The impact of glycogen addition on miRNA extraction yield. Comparison of miRNeasy, with and without the addition of glycogen. The expression of miRNAs was assessed by real-time RT-PCR. **P* <0.05, ***P* <0.01, ****P* <0.001.

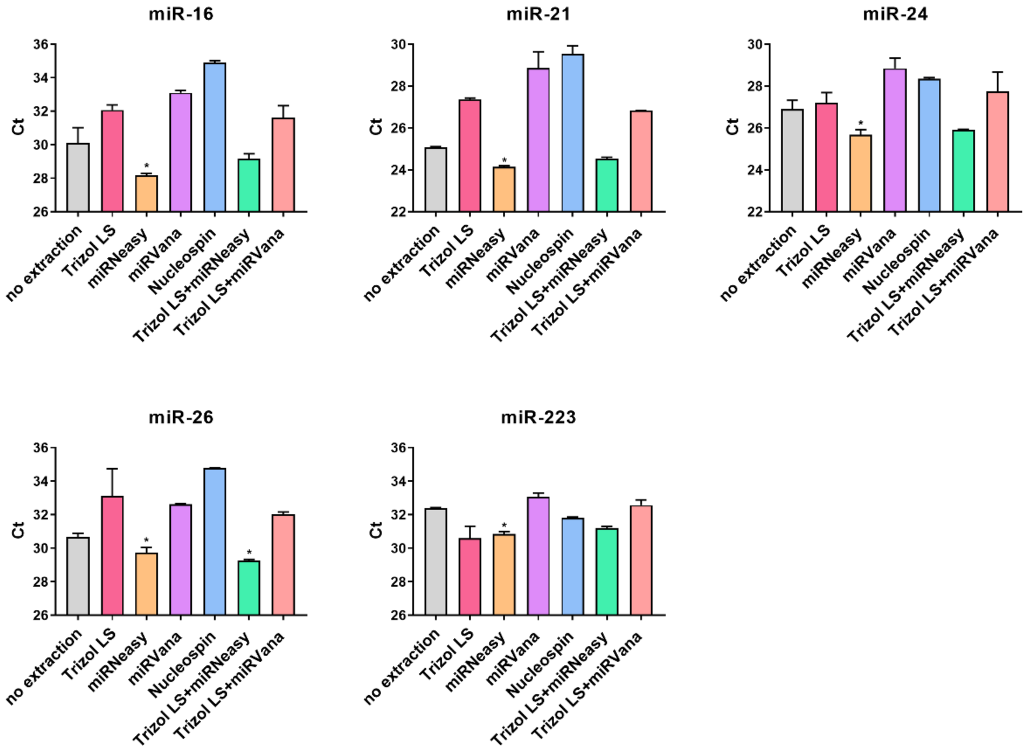

Supplementary Figure S2. Comparison of MV miRNA yield by real-time RT-PCR. The results represent average Ct values determined for five individual miRNAs. The detection of miRNA was performed by real-time RT-PCR. The mean values ± SD of three independent experiments are shown. **P* <0.05, ***P* <0.01, ****P* <0.001.

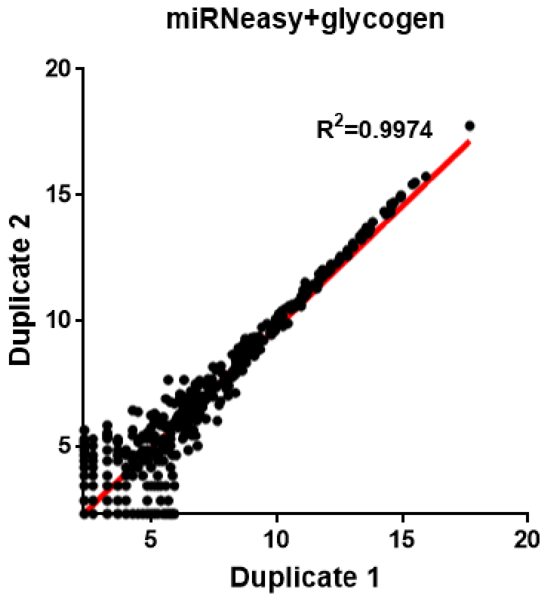

Supplementary Figure S3. Scatter plot to evaluate technical variations of the miRNeasy method.

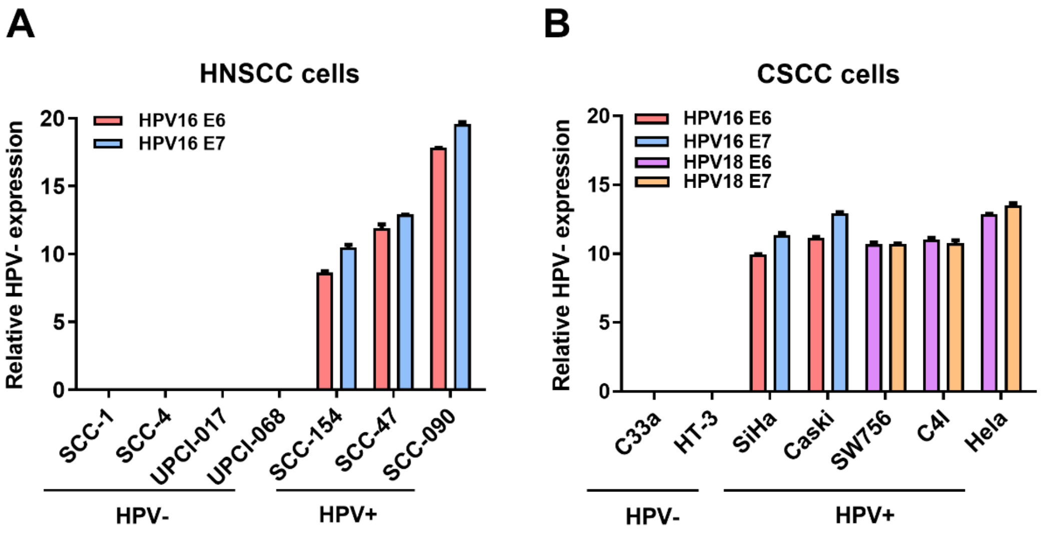

Supplementary Figure S4. The expression of HPV E6 and E7 in HNSCC cells **(A)** and CSCC cells **(B)**.

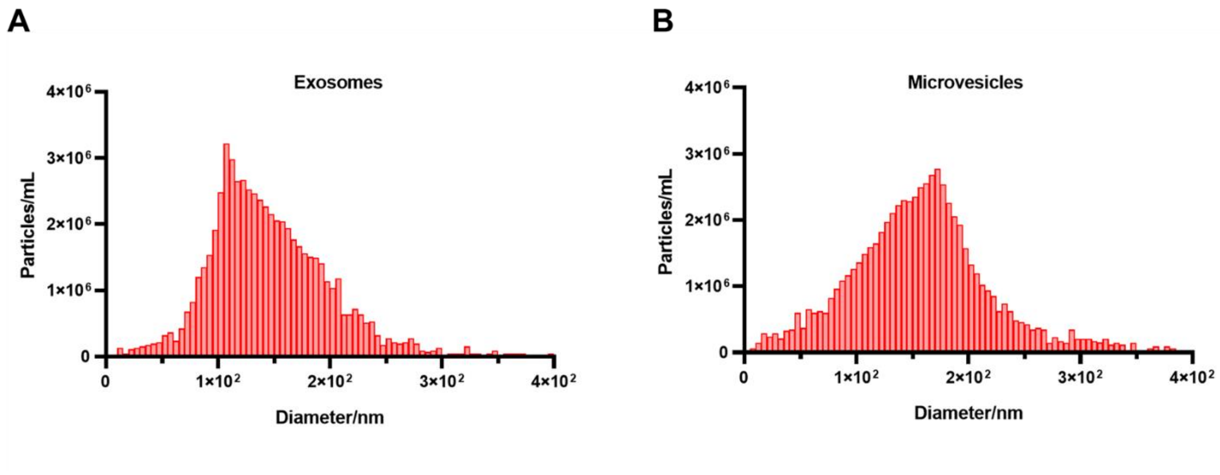

Supplementary Figure S5. Identification of EVs derived from SiHa cells. Nanoparticle tracking analysis (NTA) of exosomes (A) and microvesicles (B).
